# Individualized Wound Closure—Mechanical Properties of Suture Materials

**DOI:** 10.3390/jpm12071041

**Published:** 2022-06-25

**Authors:** Elias Polykandriotis, Jonas Daenicke, Anil Bolat, Jasmin Grüner, Dirk W. Schubert, Raymund E. Horch

**Affiliations:** 1 Department of Plastic, Hand and Microsurgery, Sana Hospital Hof, 95032 Hof, Germany; 2 Department of Plastic and Hand Surgery, University of Erlangen Medical Center, 91054 Erlangen, Germany; jasmin.gruener@uk-erlangen.de (J.G.); raymund.horch@uk-erlangen.de (R.E.H.); 3 Department of Materials Science and Engineering, Friedrich-Alexander-University Erlangen-Nürnberg, 91054 Erlangen, Germany; jonas.daenicke@fau.de (J.D.); dirk.schubert@fau.de (D.W.S.); 4 Department of Orthopedics, Theresien Hospital, 90491 Nürnberg, Germany; anil.bolat@icloud.com

**Keywords:** suture materials, crush load, mechanical properties, wound closure

## Abstract

Wound closure is a key element of any procedure, especially aesthetic and reconstructive plastic surgery. Therefore, over the last decades, several devices have been developed in order to assist surgeons in achieving better results while saving valuable time. In this work, we give a concise review of the literature and present a biomechanical study of different suturing materials under mechanical load mimicking handling in the operating theatre. Nine different suture products, all of the same USP size (4-0), were subjected to a standardized crushing load by means of a needle holder. All materials were subjected to 0, 1, 3 and 5 crushing load cycles, respectively. The linear tensile strength was measured by means of a universal testing device. Attenuation of tensile strength was evaluated between materials and between crush cycles. In the pooled analysis, the linear tensile strength of the suture materials deteriorated significantly with every cycle (*p* < 0.0001). The suture materials displayed different initial tensile strengths (in descending order: polyglecaprone, polyglactin, polydioxanone, polyamid, polypropylene). In comparison, materials performed variably in terms of resistance to crush loading. The findings were statistically significant. The reconstructive surgeon has to be flexible and tailor wound closure techniques and materials to the individual patient, procedure and tissue demands; therefore, profound knowledge of the physical properties of the suture strands used is of paramount importance. The crushing load on suture materials during surgery can be detrimental for initial and long-term wound repair strength. As well as the standard wound closure methods (sutures, staples and adhesive strips), there are promising novel devices.

## 1. Introduction

Most surgical fields are defined by an anatomical system. Visceral surgery, for instance, is the surgery of the bowel and neurosurgery is deployed in the central or peripheral nervous system. However, in the epicenter of aesthetic and reconstructive surgery lies a concept, rather than an anatomical system. It is all about reconstitution of tissue defects and functional deficits. In this task, the reconstructive surgeon is challenged by the fact that many tissue elements and suture materials have to be used in many different ways. Profound knowledge of these materials and their biomechanical properties is invaluable. There are numerous reports on surgical suturing techniques and patterns, as well as on different suture materials. Although each suture material must undergo excessive testing procedures before it is officially approved as a medical device, there are few data concerning changes in the strength and behavior of sutures during surgical handling and mechanical suture trauma. In open surgery, it is standard that only the end of the suture—that will be discarded—should be mechanically grasped to avoid weakening of the suture. However, when repeated instrument handling is necessary, such as in laparoscopic or robotic surgery, mostly resorbable sutures may well lead to a breakdown of the material, which, according to Bariol et al. [1], has not been well investigated so far [2]. It is obvious that repeated instrument handling of sutures with a needle holder might damage the surface or texture of any suture. Additional care should be taken.

Abhari et al. performed an excellent overview of the current developments in suture materials, postulating that there are still limitations in their use [3]. Until recently, there has been low academic interest in the evolution of suturing devices. Advancements were mainly industry-driven, promoting low cost and strict compliance to the regulatory setting. Polymer optimizations and antimicrobial coating were the main advances. However, suture failure is still a problem, with challenges such as knot slippage, cheese wiring, tearing of the suture through tissue when under tension remaining unsolved. Maybe the suture–tissue interface presents the single weakest link in soft tissue repair. In the near future, bioactive products will probably shift the role of suture materials from mechanical and biologically inert threads to healing-promoting devices [3].

In this work, we give a concise review of the literature and present a biomechanical study of different suturing materials under mechanical load mimicking during surgery.

## 2. Materials and Methods

### 2.1. Suture Materials

**Figure 1:**
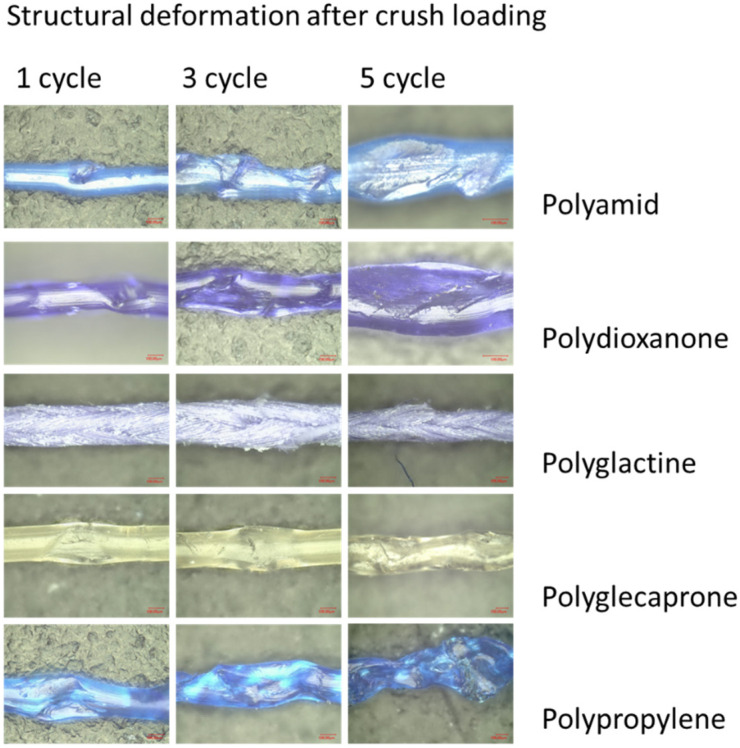
Mechanical distortion after 1, 3 and 5 crushes.

For this study, nine products were compared to each other in respect to their linear tensile strength after a standardized crushing load by a needle holder. For the sake of comparability, only threads of the size 4-0 USP (United States Pharmakopeia (UPS)) were used. A summary of the different materials used is provided in Table 1. The effect of crushing load is displayed in Figure 1. Similar study designs for comparison of suture materials have been used in the past [4]. 

### 2.2. Group Allocation

Nine different suture products were used for this line of experiments. Forty threads from every product were used for the study. In groups of 10, they were subjected to 0, 1, 3 or 5 cycles of crush loading by means of a standard needle holder (4U Medikal, Ankara, Turkey). Every one of these threads was measured and described in an experimental array later. There were 360 measurements altogether.

### 2.3. Experimental Array

A standard needle holder (4U Medikal, Ankara, Turkey) was used for all experiments. With this instrument, a crushing load was applied on the threads. The threads were crushed 0, 1, 3 or 5 times prior to measurement of the linear tensile strength. The clamp mechanism of the needle holder was used for locking the jaws on the thread. The force needed to lock the jaws of the needle holder was determined in a separate experiment, as follows.

**Figure 2:**
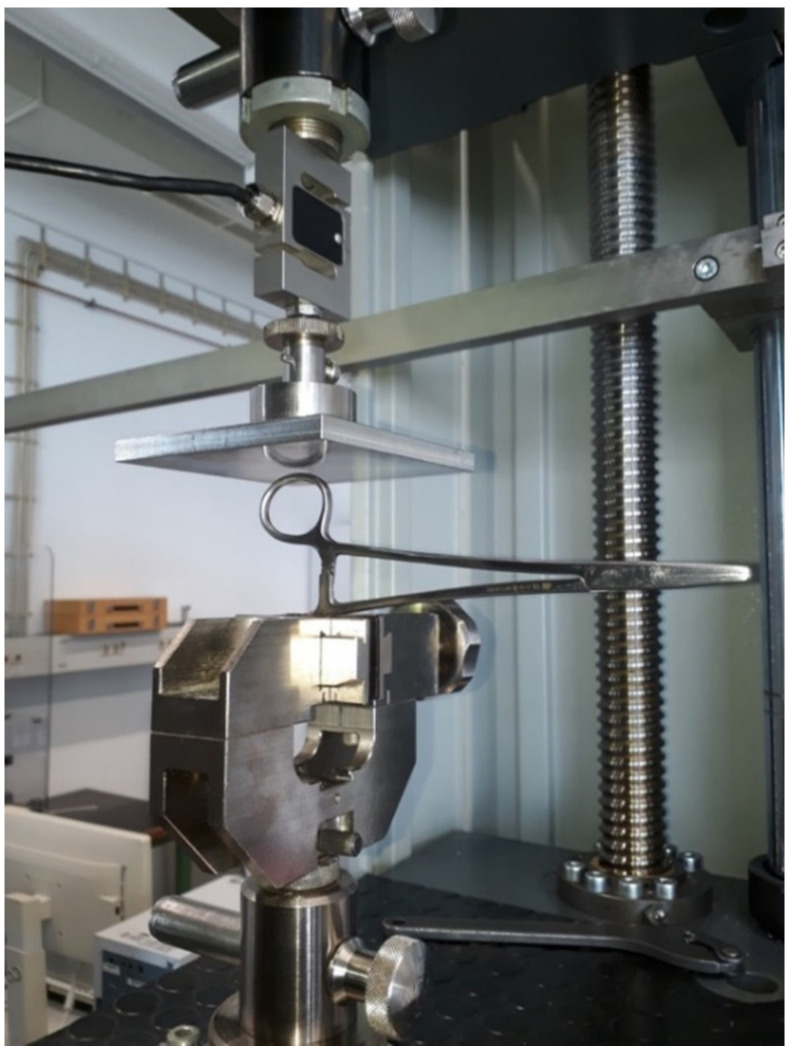
Experimental array for determination of the force (N) required to lock the jaws of the needle holder using the clamp mechanism. It was found to be 35 N.

To perform this particular measurement, the needle holder was mounted on a compression dynamometer (ZwickRoell GmbH & Co. KG, Ulm, Germany) (Figure 1). The force required to lock the instrument by compressing the clamping mechanism to its maximum (3 notches) with a 0.2 mm spacer between the jaws was found to be approximately 35 N. A thread diameter of 0.15–0.2 corresponds to USP 4-0 (Figure 2).

**Figure 3:**
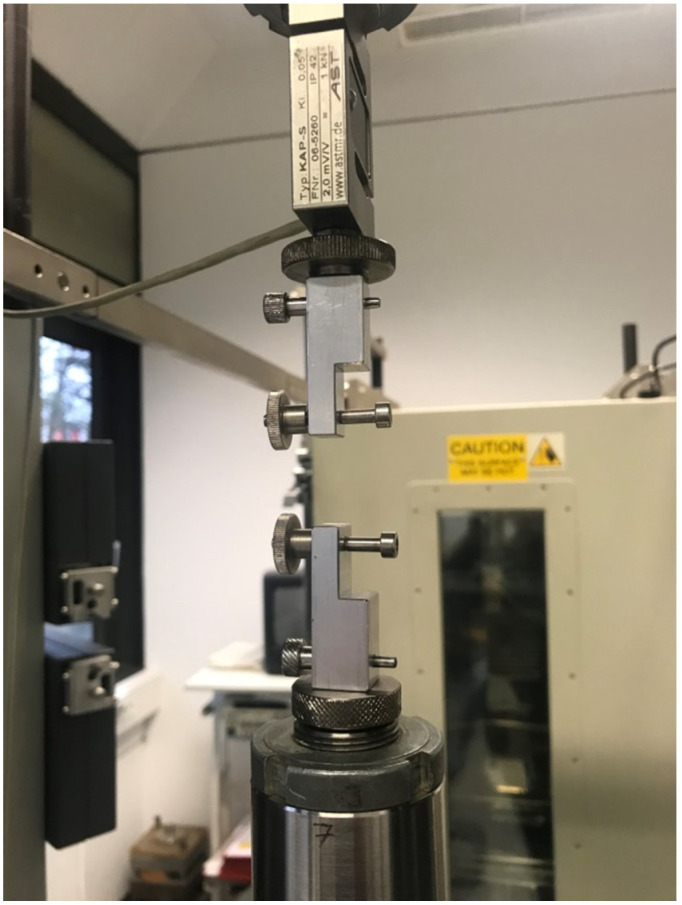
Experimental array for determination of the linear tensile strength of the suture materials.

Subsequently, for the measurements of the tensile strength of the threads, a Zwick Z050 universal dynamometer (ZwickRoell GmbH & Co. KG, Ulm, Germany) was used. For the measurement, a 100 N measuring component was mounted with a velocity setting of 300 mm/min. This method has been validated before [5,6]. The force required to evoke tear of the material was recorded. For evaluation of the results, the testXpert software (ZwickRoell GmbH & Co. KG, Ulm, Germany) was used (Figure 3).

### 2.4. Statistical Analysis

Two-way ANOVA was used for comparison between the groups using the Bonferroni correction for multiple comparisons. Parameters are generally displayed as mean values with standard deviation (±) and range.

## 3. Results

**Figure 4:**
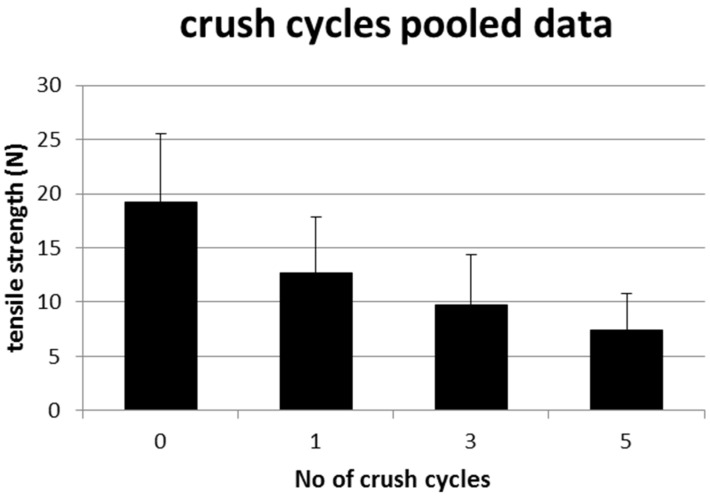
Deterioration of tensile strength of the suture materials with increasing cycles of crush load. All comparisons between the groups were highly significant (*p* < 0.0001).

The pooled mean tensile strength in the group with zero crush load was 19.21 N (±6.371, 11.66–30.72). It deteriorated to 12.70 (±5.13, 7.62–21.33) after one crush, to 9.75 (±4.63, 4.48–18.24) after three crushes and down to 7.36 (±3.45, 3.49–15.18) after five crush cycles. All comparisons were highly significant (*p* < 0.0001). These results are demonstrated in Table 2 and Figure 4.

**Figure 5:**
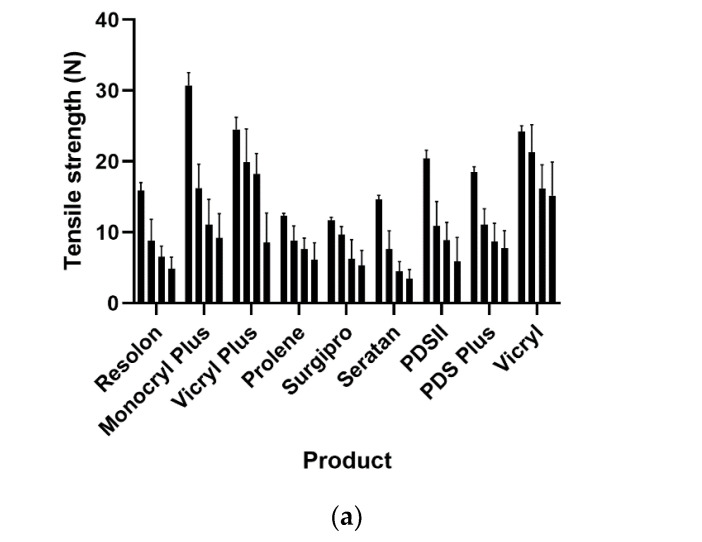

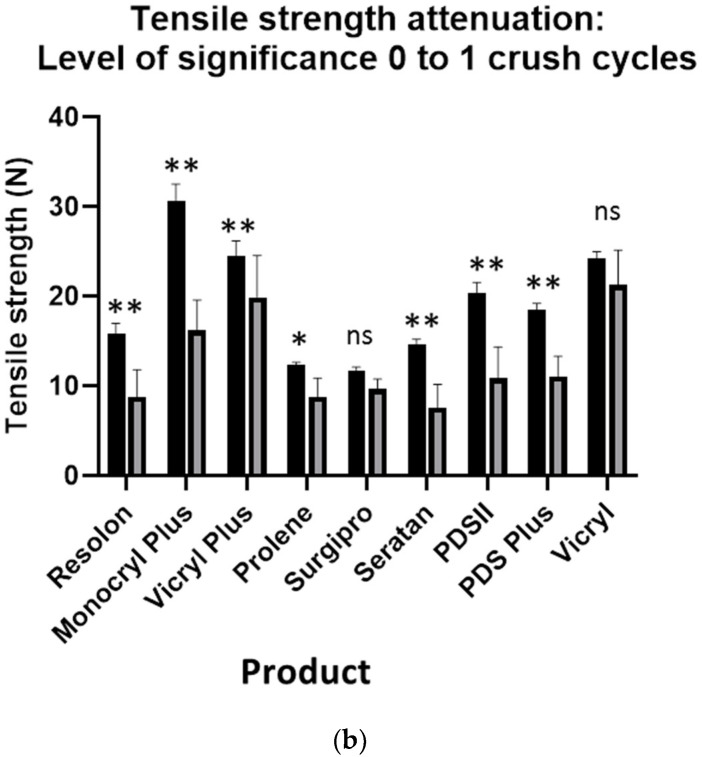
(**a**) Attenuation of tensile strength for all suture products. (**b**) Attenuation of tensile strength for all suture products between 0 and 1 crush cycles. ** = highly significant, * = significant, ns = non significant, black color: tensile strength prior to crushing load, grey color: tensile strength after one crushing load with needle-holder.

After one crush cycle, all the products displayed a significant deterioration in tensile strength, except Vicryl^®^ (*p* = 0.077) and Surgipro^®^ (*p* = 0.496). In the comparison between zero and three crush cycles, all the products showed a significant deterioration in tensile strength. The detailed results listed by product description are displayed in Table 3 and Figure 5.

**Figure 6:**
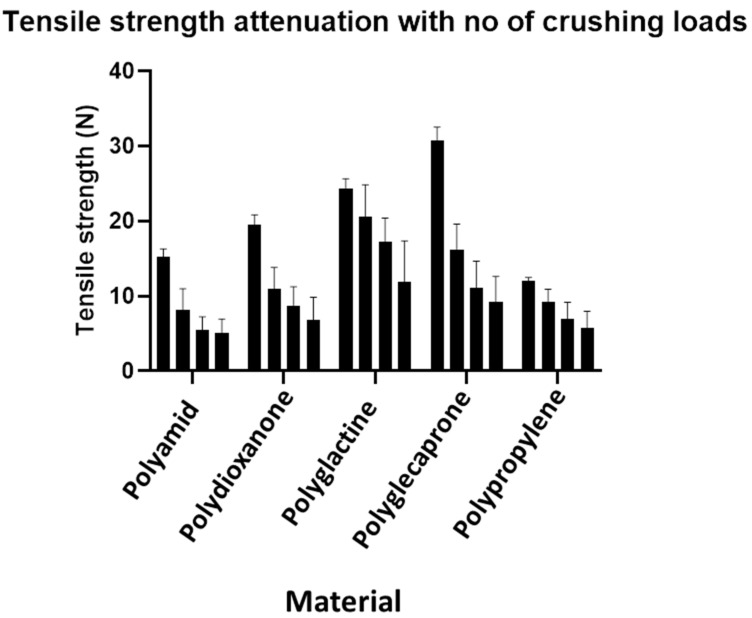
Graphical presentation of the findings in Table 4.

**Figure 7:**
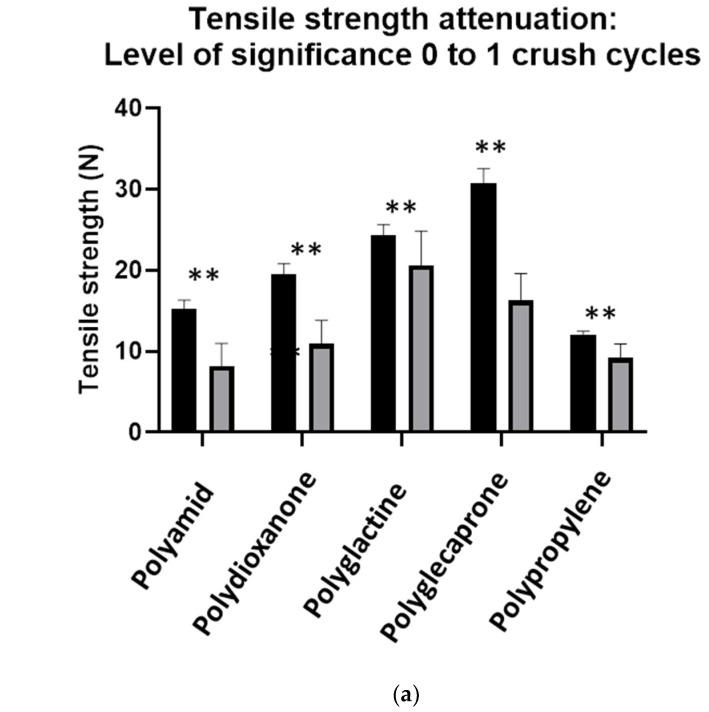

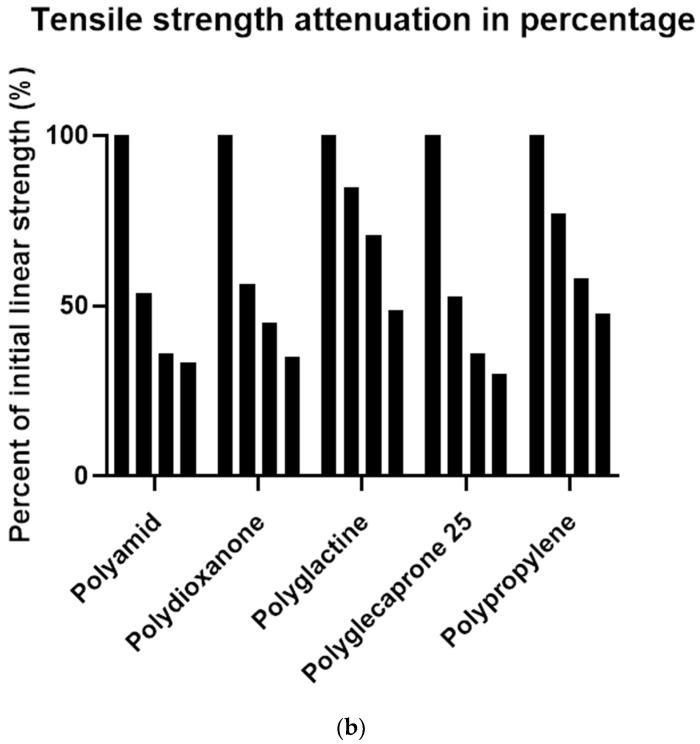
(**a**) A graphical presentation of the findings in Table 4 (partial results), black color: tensile strength prior to crushing load, grey color: tensile strength after one crushing load with needle-holder. (**b**) Attenuation of tensile strength in percent of initial linear strength. Relative values. ** = highly significant.

When analyzed by material, all the comparisons after one or three crush cycles were significant. The corresponding results are summarized in Table 4 (descriptive), Table 5 (inferential statistics) and Figure 6 and Figure 7.

## 4. Discussion

Sutures are the mainstay of any surgical procedure, either as an interrupted or continuously applied closure method. Generally, the physical properties of sutures, such as the suture diameter, tensile strength, elongation, surface roughness, coefficient of friction, bending stiffness and tissue drag, and the knot characteristics are well characterized and have been studied extensively [1]. Such tests are, in fact, an integral part of the regulatory procedures before a suture is officially approved as a medical device [7]. However, only a few studies investigated the mechanical damage to sutures by repeated instrument handling during surgery [8]. According to Naleway et al., most published reports on the tensile behavior of various sutures only focus on breaking force, and detailed reports comparing other important tensile properties, such as failure elongation, failure stress, failure strain, modulus, and full stress–strain curves across suture materials, are quite limited [9]. Most researchers agree that simple knotting alone influences materials that may differ significantly in their tensile strengths and elastic/plastic deformation characteristics, but can still display comparable elongations at failure [6]. Von Fraunhofer et al. [10] described that all sutures in their study showed decreased tensile strength and elongation at failure when knotted. Most of their investigated materials showed increased tensile strength and decreased elongation at failure for smaller suture gauges (thicker strands), and this behavior is thought to be related to their internal molecular organization [11].

Clamp fixation for preventing the unfolding of a suture knot has been described to weaken the tensile strength of polypropylene sutures by Türker et al. [12]. Studies on the grip forces regarding hand and finger movements or force control reflected by individual grip force data may help to gain further data on dominant and nondominant hand influences, but do not apply in this context as the needle holder used here offered a distinct and reliable predetermined force when operated [13]. In laparoscopic and robotic surgery, it has been noted that the lack of haptic feedback has become a growing issue due to the application of excessive force that may lead to clinical problems, such as intraoperative and postoperative suture breakage [11]. Latest sensing technology and haptic feedback systems that can reduce instances of suture failure without negatively impacting performance outcomes, including knot quality, are, therefore, under investigation [11,14,15].

In these studies [11,14], it was also pointed out that the loop created in any suture is most prone to failure due to suture elongation, knot slip and suture breakage. It was found that monofilament sutures offered higher bending stiffness, but also a higher tendency to untie, compared to monofilament sutures. Interestingly, the ultimate failure load of monofilament nonabsorbable polypropylene sutures (Prolene) was significantly reduced when compared to the ultimate failure load achieved by other monofilament sutures, such as polyglyconate and nylon, as well as braided absorbable polyglactin, which were not affected by correcting the first throw of the loop, in their experimental array [12]. Mechanical grip of sutures with a clamp or needle holder is often applied when sutures have to be tied under tension to temporarily secure a knot after the first throw, to minimize unwanted gap formation until further throws are performed and fix the whole knots. Bisson et al. found that ForceFiber suture loops tied with serrated clamps were reduced by approximately 21% compared to those tied with no clamp (227 N vs. 289 N, *p* = 0.003), and approximately 18% compared to those tied with a smooth clamp [16].

In open surgery, it is a rule that only the end of the suture—that will be discarded —should be mechanically grasped to avoid weakening of the suture. However, since laparoscopic and robotic surgery has become a frequent and standard approach for many indications, the problem of suture trauma by instrument handling becomes an important issue. It has been shown that sutures in robot-assisted vascular surgery showed a significant loss of strength. Surgeons have, therefore, begun to search for materials that are most resistant against robotic mechanical handling. The properties of different sutures have been investigated to find out which materials are least susceptible to robotic manipulations and, therefore, could best be considered as materials of first choice [17].

Laparoscopic surgery necessitates the mechanical manipulation of sutures due to the limited access to the operative field. According to Bariol et al., absorbable sutures are especially bound to suffer material break-down upon repeated instrument handling. This effect has not been well investigated so far [1]. In continuous sutures, a broken suture can result in loss of achieved tissue approximation and lead to significant clinical problems. It is obvious that repeated instrument handling of sutures with a needle holder might alter the surface or texture of any suture. Until now—apart from anecdotal and rare experimental reports—it remained unclear how precisely sutures are damaged by doing so and how many grips it takes to ruin a suture. The recommendation of Bisson and coworkers, which was that temporarily clamping a knot to keep it from slipping during the tying process when securing sutures can be performed without any concern for weakening the suture or without imposing the danger of suture trauma [16], cannot be confirmed by our findings. Our experimental set up mimicked the clinical situation in a standardized way and clearly revealed the increasing damage to the various sutures with the increasing number of clamping procedures. Here, we could show, for the first time, exactly how repeated clamping with a needle holder alters the surface and texture of various suture materials and reduces their breaking strength. 

Other studies that intended to define the breaking strength by repeated elongation of sutures are not directly comparable to our experiments. Dobrin and Mrkvicka found that chronic loading of polypropylene sutures increased their “acute” breaking force. They suggested that this may have resulted from increased orientation of crystals in the core of the filaments [8,18]. Dobrin et al. briefly applied stress to suture materials by disturbing the outer surface of the filament by placing a stray knot or pinching with forceps, which both resulted in a decreased “acute” breaking strength [19].

In this study, we did not tackle the challenges posed by the use of different suture–needle configurations and handling of the needle itself. Here, surgical skills are of paramount importance for adequate tissue repair [20]. One should be aware of the different indications of tapered, round, cutting and reversed cutting needle designs. Apart from that, some of the alloys (S45500) [21] used to manufacture the needles contain a substantial proportion of nickel, rendering them unsuitable for patients with a nickel allergy. Finally, grasping the needle at the “eye” weakens the link to the suture material. 

As expected, our experiment confirmed that a single crush with the needle holder can significantly reduce the tensile strength of a suture strand by approximately 30%. However, when investigated separately, not all the products displayed the same effect. In particular, Vicryl^®^ (polyglactine) and Surgipro^®^ (polypropylene) suffered non-significant material damage in terms of linear strength. In fact, braided polyglactine (Vicryl^®^) showed resistance to crush load and lost the least linear tensile strength upon deformation. Polypropylene possessed the least initial linear tensile strength, but strength deterioration was relatively low. When the strands were investigated by material (and not as a separate product), a single cycle of crush load in all cases produced significant attenuation of linear tensile strength. There was no marked or significant difference between resorbable and non-resorbable materials, although the study design was inappropriate for this question.

Other newer technologies, such as staples, various skin adhesives, zipper-like devices, and polyester meshes, have gained popularity in the operating theatre [22]. Although adverse reactions to the adhesives [23] or to nickel alloys in metallic staplers have been reported, these new devices definitely have a place in orthopedic [24] and cardiovascular surgery [25], but are yet to acquire wide acceptance in plastic surgery. This might change with the development of novel degradable polyurethane-based tissue sealants [26]. Numerous reports on wound closure after knee surgery showed that zipper-like devices can produce equal or better results than staples [27]. Finally, the use in ointments and gels loaded with biomodulatory molecules [28] is yet to be fully evaluated, but seems to be an option in burns and diabetic foot ulcers. Products containing basic and acidic fibroblastic growth factor (bFGF and aFGF), granulocyte macrophage colony-stimulating growth factor (GM-CSF), platelet-derived growth factor (PDGF) as well as epidermal growth factor (EGF) are available. However, some concerns arose following reports of malignancies emerging in combination with the use of recombinant PDGF gels (becaplermin-Regranex^®^) [29,30].

## 5. Conclusions

Repeated instrumental handling of various suture materials inevitably leads to mechanical damage of sutures and premature break down, even with perfectly knotted sutures. Especially in continuous sutures, this can have severe clinical consequences. Our data clearly reveal the deleterious effect on sutures when they are repeatedly grasped with an instrument during knotting. It, therefore, seems advisable to take meticulous care without unnecessary grasping of the material. Reconstructive surgeons have to be flexible and tailor their wound closure techniques and materials to individual patients, as well as to the procedure and tissue demands; therefore, profound knowledge of the physical properties of the suture materials used is of paramount importance.

## Figures and Tables

**Table 1:** A list of the used suture threads. All threads were 4-0 USP.

Product	Manufacturer	Material	Type	Absorbable	Coating
Vicryl^®^ Plus	Ethicon	Polyglactine 910	Braided	+	+
Vicryl^®^	Ethicon	Polyglactine 910	Braided	+	−
Monocryl^®^ Plus	Ethicon	Polyglecaprone 25	Braided	+	+
PDS^®^ Plus	Ethicon	Polydioxanone	Monofil.	+	+
PDS^®^ II	Ethicon	Polydioxanone	Monofil.	+	−
Prolene^®^	Ethicon	Polypropylene	Monofil.	−	−
Surgipro^®^	Covidien	Polypropylene-Polyethylene	Monofil.	−	−
Seratan^®^	Serag-Wiessner	Polyamide	Monofil.	−	+
Resolon^®^	Resorba	Polyamide	Monofil.	−	−

Coating for Vicryl plus, Monocryl plus and PDS plus is a broad spectrum antibiotic (Triclosan), coating for Seratan is Titanium. Monofil. = monofilamentous. + = with, − = without.

**Table 2:** Crush cycles, pooled data over all suture materials. All comparisons displayed high statistical significance.

Crushing Load (Cycles)	Mean Tensile Strength (N)	Tensile Strength Remaining (%)
0	19.21 (±6.371, 11.66–30.72)	100.0
1	12.70 (±5.13, 7.62–21.33)	66.1
3	9.75 (±4.63, 4.48–18.24)	50.0
5	7.36 (±3.45, 3.49–15.18)	38.3

**Table 3:** Tensile strength deterioration detailed for all suture products.

Product	0 Crushing Load		1× Crushing Load		3× Crushing Load		5× Crushing Load	
	Mean	(SD)	Mean	(SD)	Mean	(SD)	Mean	(SD)
Resolon	15.87	(1.13)	8.78	(3.03)	6.50	(1.55)	4.86	(1.63)
Monocryl Plus	30.72	(1.82)	16.24	(3.37)	11.06	(3.60)	9.19	(3.43)
Vicryl Plus	24.52	(1.69)	19.91	(4.66)	18.24	(2.88)	8.55	(4.15)
Prolene	12.30	(0.35)	8.80	(2.09)	7.60	(1.59)	6.10	(2.43)
Surgipro	11.66	(0.44)	9.65	(1.14)	6.28	(2.67)	5.33	(2.12)
Seratan	14.62	(0.59)	7.62	(2.56)	4.48	(1.35)	3.49	(1.23)
PDSII	20.43	(1.16)	10.85	(3.49)	8.86	(2.51)	5.87	(3.37)
PDS Plus	18.53	(0.72)	11.08	(2.25)	8.64	(2.61)	7.74	(2.49)
Vicryl	24.22	(0.78)	21.33	(3.85)	16.17	(3.33)	15.18	(4.75)

**Table 4:** Tensile strength deterioration detailed for all suture materials.

Material	0 Crushing Load		1× Crushing Load		3× Crushing Load		5× Crushing Load	
	Mean (SD)	Remaining Linear Strength	Mean (SD)	Remaining Linear Strength	Mean (SD)	Remaining Linear Strength	Mean (SD)	Remaining Linear Strength
Polyamid	15.25 (1.09)	100%	8.20 (2.80)	53.79%	5.49 (1.75)	36.01%	5.10 (1.85)	33.42%
Polydioxanone	19.48 (1.35)	100%	10.97 (2.86)	56.29%	8.75 (2.49)	44.92%	6.81 (3.04)	34.93%
Polyglactine	24.37 (1.29)	100%	20.62 (4.22)	84.61%	17.21 (3.21)	70.60%	11.87 (5.51)	48.69%
Polyglecaprone	30.72 (1.82)	100%	16.24 (3.37)	52.86%	11.06 (3.60)	36.00%	9.19 (3.43)	29.92%
Polypropylene	11.98 (0.51)	100%	9.23 (1.70)	77.00%	6.94 (2.24)	57.93%	5.72 (2.25)	47.70%

**Table 5:** Analysis of the effect of 0 to 5 crush loading cycles on the linear tensile strength of different suture materials (two-way ANOVA with Bonferroni correction for multiple comparisons.

Material	0× vs. 1× Crushing Load	1× vs. 3× Crushing Load	3× vs. 5× Crushing Load
	Level of Significance	Level of Significance	Level of Significance
Polyamid	*p* < 0.0001 (**)	*p* = 0.0110 (*)	*p* > 0.9999 (ns)
Polydioxanone	*p* < 0.0001 (**)	*p* = 0.0639 (ns)	*p* = 0.1486 (ns)
Polyglactine	*p* = 0.0001 (**)	*p* = 0.0005 (**)	*p* < 0.0001 (**)
Polyglecaprone	*p* < 0,0001 (**)	*p* = 0.0002 (**)	*p* = 0.7571 (ns)
Polypropylene	*p* = 0.0092 (**)	*p* = 0.0507 (ns)	*p* = 0.9387 (ns)

** = highly significant, * = significant, ns = non significant.

## Data Availability

Detailed data supporting the results are available with the authors.
